# Ranking hospitals when performance and risk factors are correlated: A simulation-based comparison of risk adjustment approaches for binary outcomes

**DOI:** 10.1371/journal.pone.0225844

**Published:** 2019-12-04

**Authors:** Martin Roessler, Jochen Schmitt, Olaf Schoffer

**Affiliations:** Zentrum für Evidenzbasierte Gesundheitsversorgung, Universitätsklinikum und Medizinische Fakultät Carl Gustav Carus an der Technischen Universität Dresden, Dresden, Germany; Tongii University, CHINA

## Abstract

**Background:**

The conceptualization of hospital quality indicators usually includes some form of risk adjustment to account for hospital differences in case mix. For binary outcome variables like in-hospital mortality, frequently utilized risk adjusted measures include the standardized mortality ratio (SMR), the risk standardized mortality rate (RSMR), and excess risk (ER). All of these measures require the estimation of expected hospital mortality, which is often based on logistic regression models. In this context, an issue that is often neglected is correlation between hospital performance (e.g. care quality) and patient-specific risk factors. The objective of this study was to investigate the impact of such correlation on the adequacy of hospital rankings based on different measures and methods.

**Methods:**

Using Monte Carlo simulation, the impact of correlation between hospital care quality and patient-specific risk factors on the adequacy of hospital rankings was assessed for SMR/RSMR, and ER based on logistic regression and random effects logistic regression. As an alternative method, fixed effects logistic regression with Firth correction was considered. The adequacies of the resulting hospital rankings were assessed by the shares of hospitals correctly classified into quintiles according to their true (unobserved) care qualities.

**Results:**

The performance of risk adjustment approaches based on logistic regression and random effects logistic regression declined when correlation between care quality and a risk factor was induced. In contrast, fixed-effects-based estimations proved to be more robust. This was particularly true for fixed-effects-logistic-regression-based ER. In the absence of correlation between risk factors and care quality, all approaches showed similar performance.

**Conclusions:**

Correlation between risk factors and hospital performance may severely bias hospital rankings based on logistic regression and random effects logistic regression. ER based on fixed effects logistic regression with Firth correction should be considered as an alternative approach to assess hospital performance.

## Introduction

Hospital quality indicators are used to assess and compare hospital performance. To fulfill these purposes, quality indicators should provide an adequate ranking of hospitals with respect to their (unobserved) care quality. Such assessments have become increasingly important, e.g. as a basis for the initiation of quality assurance measures [1, 2]. Moreover, hospital rankings are subject to growing public attention [3–6]. Against that background, an adequate estimation of hospital performance is of high relevance. This task is complicated by the fact that hospitals may differ with respect to risk factors like the age structure of or comorbidities in patients. To account for this issue, the conceptualization of quality indicators often includes some form of risk adjustment.

In this context, the standardized mortality ratio (SMR) is a frequently utilized measure based on indirect standardization [7, 8]. The SMR is defined as the relation between the observed mortality rate *O* and the expected mortality rate *E*, i.e. SMR = *O*/*E*. While *O* can be derived directly from the data, *E* depends on the distribution of the relevant risk factors between the hospitals and must be estimated. In practice, estimation of expected mortality is often based on logistic regression [7]. A main critique of the logistic-regression-based SMR is that it does not sufficiently account for random variation in low-volume hospitals [9]. As a result, the SMR of these hospitals is driven to extreme values. This issue is addressed by shrinkage estimators like the Risk Standardized Mortality Rate (RSMR), which is, for instance, used by the Centers for Medicare & Medicaid Services (CMS) [10]. The RSMR is also based on a model with logistic link function but additionally includes a random intercept at the hospital level. This random intercept represents hospital-specific differences in mortality and thus accounts for the hierarchical structure of the data by allowing for correlation between the outcomes of patients admitted in the same hospital. In addition to differences between hierarchical and non-hierarchical modeling, researchers and practitioners should be aware of potential problems related to the concept of the SMR itself. Since SMR is a relative measure, small absolute differences can lead to high standardized rates. An alternative measure that avoids this issue is the excess risk, which is defined as the difference between the observed and expected mortality rate, i.e. ER = *O* − *E*. Given the drawbacks of SMR, ER was recommended by some authors for indirect standardization [11].

Although these different approaches to risk adjustment were examined in multiple studies [12–15], there is a lack of evidence with respect to their performance under many relevant scenarios. In this regard, an aspect that is often neglected is correlation between hospital performance and risk factors. Such correlation may arise when hospitals with better performance treat sicker patients than hospitals with worse performance (or vice versa). This correlation may also arise if the risk adjustment model includes comorbidities without considering whether they had been present on admission (POA). If some comorbidities were caused by a low hospital-care quality, there is correlation between comorbidity indicators and unobserved hospital performance. This problem is of high practical relevance since many official data sources used to construct quality indicators, including the German Diagnoses-Related Groups Statistics [16] do not indicate whether a specific comorbidity had been POA.

Against that background, the objective of this paper was to investigate the adequacy of different approaches to risk adjustment in the presence of correlation between hospital performance and risk factors. By the example of logistic regression for a binary outcome, we highlight that common approaches implicitly rely on the assumption that risk factors and hospital performance are uncorrelated. Hence, violations of this assumption may bias the hospital performance assessment. As an alternative method that allows for such correlation, we considered fixed effects logistic regression with Firth correction [17]. Furthermore, we compared the results of SMR/RSMR-based and ER-based assessments. The performance of these approaches was examined using Monte Carlo simulations.

## Materials and methods

Note that this study focuses on hospital performance in terms of care quality. In the following, the terms hospital performance and care quality are therefore generally used interchangeably.

### Data generation process

**Fig 1:**
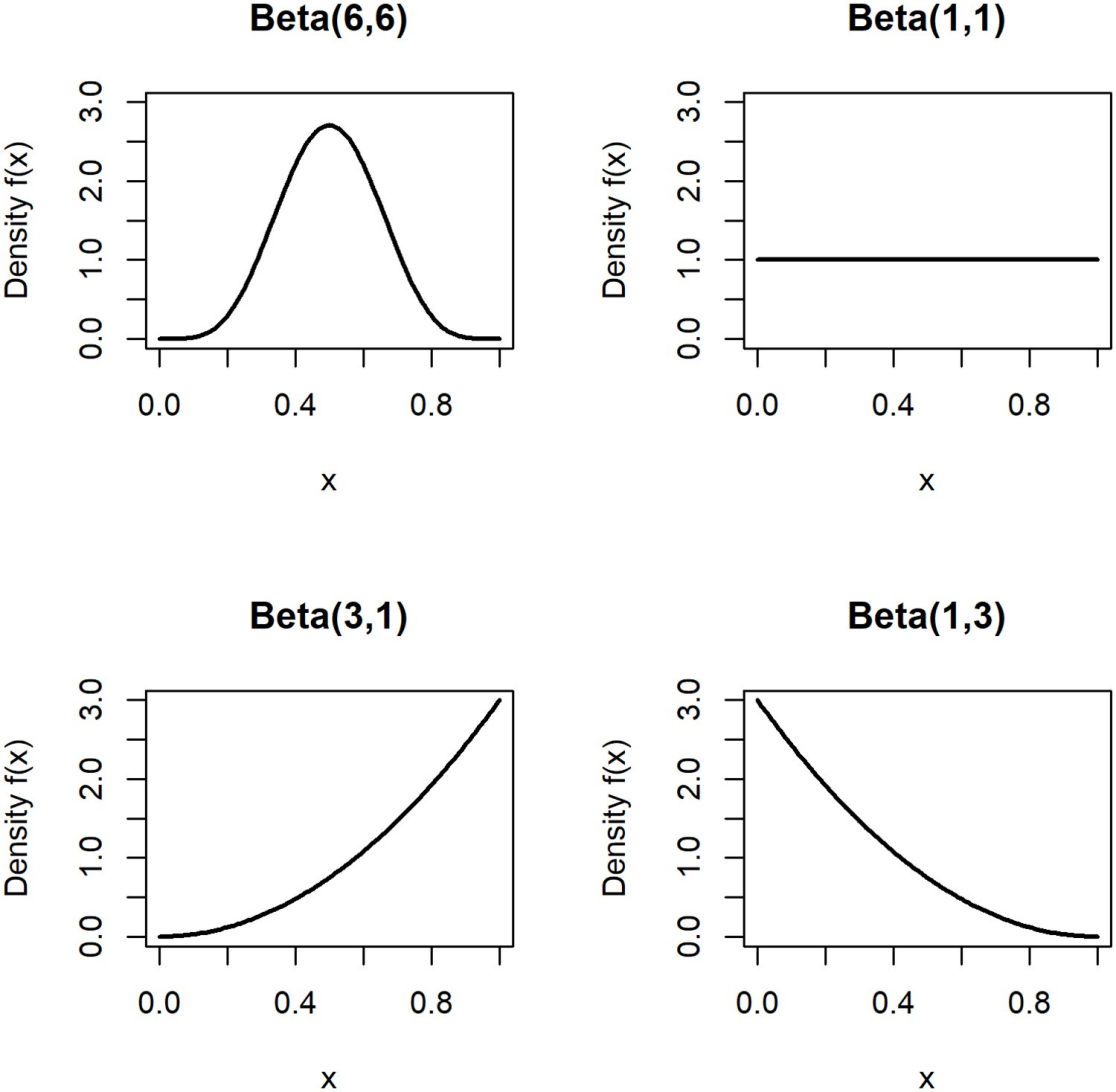
Scenarios for the distribution of hospital care quality.

We considered a binary outcome variable $Y_{hi}∼\text{Ber}\left(p_{hi}^{Y}\right)$, (e.g. mortality), where *h* = 1, …, *H* denotes hospitals and *i* = 1, …, *n*_*h*_ denotes the patients treated within a specific hospital. The probability $p_{hi}^{Y}$ of observing *Y*_*hi*_ = 1 (e.g. in-hospital death) was determined by the (unobserved) hospital-specific quality of care *Q*_*h*_ and a patient-specific risk factor *X*_*hi*_ according to

$$p_{hi}^{Y}=P\left(Y_{hi}=1|Q_{h},X_{hi}\right)=F^{-1}\left(\beta_{0}+\beta_{1}Q_{h}+X_{hi}\right),$$ (1)

where *F*(⋅) is the logistic link function and *β*_0_ and *β*_1_ are fixed coefficients. We assumed that the quality of care follows a Beta distribution, i.e. $Q_{h}\overset{i.i.d.}{∼}\text{Beta}\left(q_{1},q_{2}\right)$. Depending on the parameters *q*_1_ and *q*_2_, this gave us the opportunity to consider scenarios with symmetric and skewed quality distributions (see Fig 1).

Importantly, the data generation process allowed *Q*_*h*_ and *X*_*hi*_ to be correlated. This was achieved by specifying the generating equation for *X*_*hi*_ as

$$X_{hi}=\gamma \cdot Q_{h}+a_{h}+\varepsilon_{hi},$$ (2)

where $a_{h}\overset{i.i.d.}{∼}N\left(0,\eta^{2}\right)$ is a normally distributed variable that induces hospital-specific differences with respect to the distribution of the risk factor and $\varepsilon_{hi}\overset{i.i.d.}{∼}N\left(0,\sigma^{2}\right)$ is a normally distributed patient-specific random term. The coefficient *γ* relates *X*_*hi*_ to *Q*_*h*_ and, therefore, induces positive (negative) association between these variables if *γ* > 0 (*γ* < 0). If *γ* = 0, care quality and risk factor are independent. Note that *γ* does not have to be interpreted as inducing a causal effect but more generally determines the sign and strength of the correlation *ρ* = Corr[*X*_*hi*_, *Q*_*h*_]. In each simulated scenario, we chose a specific value of *ρ* and used the relation

$$\gamma =\text{sign}\left(\rho \right)\cdot \sqrt{\frac{\rho^{2}}{1-\rho^{2}}\cdot \left(\eta^{2}+\sigma^{2}\right)\cdot \frac{{\left(q_{1}+q_{2}\right)}^{2}\left(1+q_{1}+q_{2}\right)}{q_{1}q_{2}}}$$ (3)

to determine the value of *γ*.

It is noteworthy that correlation between risk factor and care quality influences the population average mortality rate *p* = *E*[*Y*_*hi*_]. Since this parameter may be crucial for the detection of performance differences between hospitals, we used a Taylor series approximation of Eq 1 to choose *β*_0_ n order to fix *p* at a specific value (details are provided in the supporting information S1). Another parameter that affects the chances of detecting differences in hospital performance is the effect size of care quality on the outcome *β*_1_. Given the logistic model, we set *β*_1_ = ln(*OR*), where *OR* denotes the odds ratio of mortality for the highest possible care quality (*Q*_*h*_ = 1) relative to the lowest possible care quality (*Q*_*h*_ = 0).

**Fig 2:**
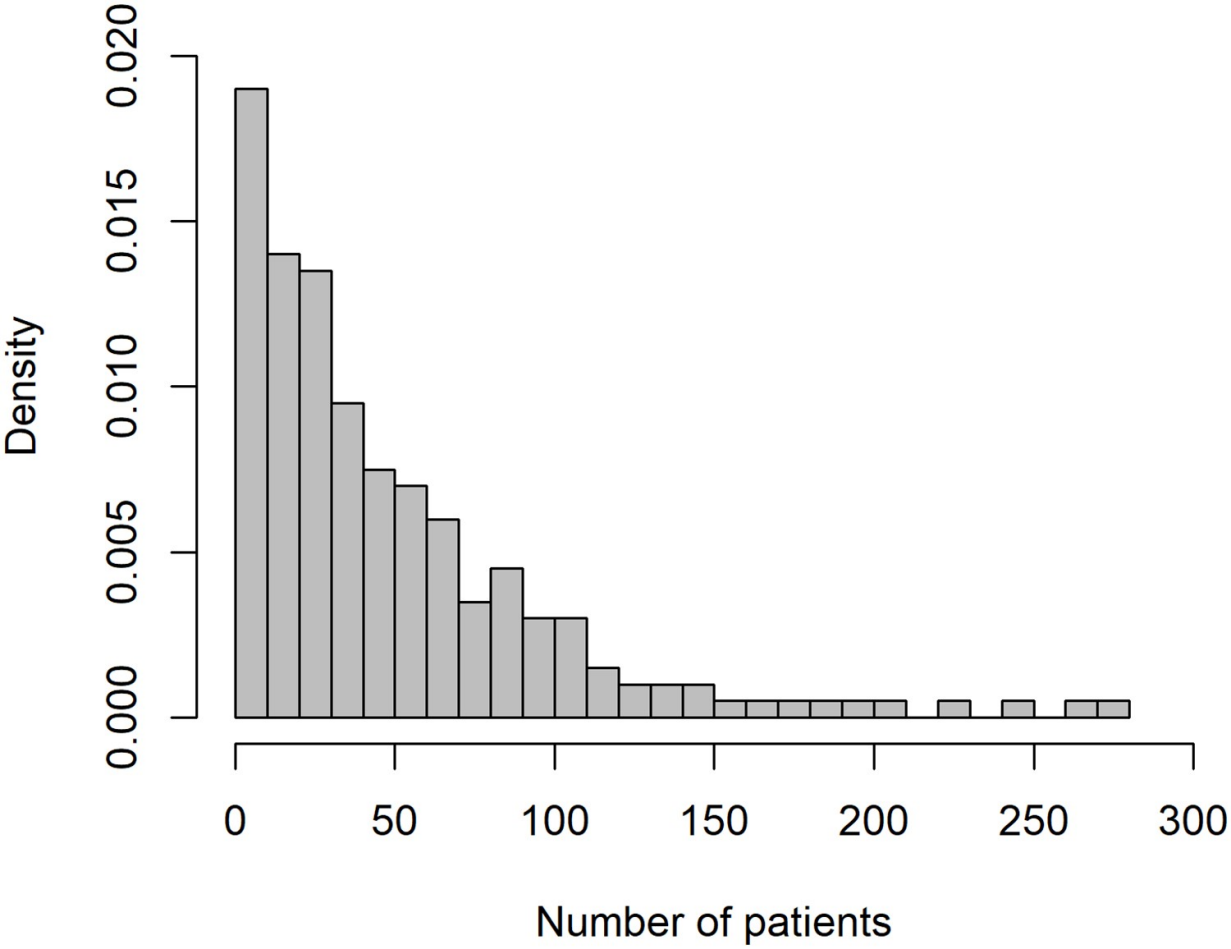
Distribution of the number of patients (*n* = 10, 000; *H* = 200).

Since datasets used for assessments of hospital performance usually include hospitals of different size, we simulated the number of patients treated in the hospitals according to the distribution of bed sizes reported in the German Hospital Directory 2016 provided by the German Federal Statistical Office [18] (see Fig 2). As outlined in detail below, the other parameters included in the data generation process were chosen such that the simulated datasets reflected properties (e.g. average mortality rates) comparable to real-world hospital data used for risk adjustment [1].

### Methods and measures

In the following, the statistical models and measures compared in this study are described. All considered models were estimated using the maximum likelihood method [19–23].

#### a1) SMR based on logistic regression

Given data on outcome *Y*_*hi*_ and risk factor *X*_*hi*_, one approach relied on the estimation of the logistic regression model

$$E\left[Y_{hi}|X_{hi}\right]=F^{-1}\left(\alpha_{0}+\alpha_{1}X_{hi}\right).$$ (4)

Based on the parameter estimates ${\hat{\alpha }}_{0}$ and ${\hat{\alpha }}_{1}$, patient-specific predicted probabilities of death were calculated as ${\hat{p}}_{hi}^{Y}=F^{-1}\left({\hat{\alpha }}_{0}+{\hat{\alpha }}_{1}X_{hi}\right)$. The mean of these probabilities across all patients treated in a hospital then served as the expected mortality rate for this hospital, i.e. ${\hat{E}}_{h}^{\text{Logit}}=n_{h}^{-1}\sum_{i=1}^{n_{h}}{\hat{p}}_{hi}^{Y}$. The logistic regression-based SMR therefore is given by

$${\hat{\text{SMR}}}_{h}^{\text{Logit}}=\frac{O_{h}}{{\hat{E}}_{h}^{\text{Logit}}}=\frac{\sum_{i=1}^{n_{h}}Y_{hi}}{\sum_{i=1}^{n_{h}}F^{-1}\left({\hat{\alpha }}_{0}+{\hat{\alpha }}_{1}X_{hi}\right)},$$ (5)

where $O_{h}=n_{h}^{-1}\sum_{i=1}^{n_{h}}Y_{hi}$ is the observed mortality rate of hospital *h*.

#### a2) ER based on logistic regression

As an alternative to the SMR derived from the logistic regression model, the logistic-regression-based excess risk is defined as ${\hat{\text{ER}}}_{h}^{\text{Logit}}=O_{h}-{\hat{E}}_{h}^{\text{Logit}}$.

#### b1) RSMR based on random effects logistic regression

Following the methodology of CMS [10], the calculation of the RSMR was based on the hierarchical logistic regression model

$$E\left[Y_{hi}|X_{hi}\right]=F^{-1}\left(\alpha_{0h}+\alpha_{1}X_{hi}\right),$$ (6)

where $\alpha_{0h}\overset{i.i.d.}{∼}N\left(\mu ,\xi^{2}\right)$ is a hospital-specific, normally distributed term with mean *μ* and variance *ξ*^2^. Since *α*_0*h*_ is a random intercept, Eq 6 represents a random effects (RE) model. The random effects RSMR further differs from the logistic-regression-based SMR as it does not relate the observed mortality rate to the expected mortality rate. Rather, it considers the expected mortality rate of a hospital conditional on its estimated performance level ${\hat{\alpha }}_{0h}$, i.e. ${\hat{E}}_{h}^{\text{RE}\;\text{Logit}}=n_{h}^{-1}\sum_{i=1}^{n_{h}}F^{-1}\left({\hat{\alpha }}_{0h}+{\hat{\alpha }}_{1}X_{hi}\right)$, relative to its expected mortality rate conditional on the estimated average hospital performance level $\hat{\mu }$, i.e. ${\hat{\bar{E}}}_{h}^{\text{RE}\;\text{Logit}}=n_{h}^{-1}\sum_{i=1}^{n_{h}}F^{-1}\left(\hat{\mu }+{\hat{\alpha }}_{1}X_{hi}\right)$. The estimate of the random effects RSMR thus is

$${\hat{\text{RSMR}}}_{h}^{\text{RE}}=\frac{{\hat{E}}_{h}^{\text{RE}\;\text{Logit}}}{{\hat{\bar{E}}}_{h}^{\text{RE}\;\text{Logit}}}=\frac{\sum_{i=1}^{n_{h}}F^{-1}\left({\hat{\alpha }}_{0h}+{\hat{\alpha }}_{1}X_{hi}\right)}{\sum_{i=1}^{n_{h}}F^{-1}\left(\hat{\mu }+{\hat{\alpha }}_{1}X_{hi}\right)}.$$ (7)

Since the expected mortality ${\hat{E}}_{h}^{\text{RE}\;\text{Logit}}$ lies in between 0 and 1, the RSMR-values of small hospitals are to some extent shrinked towards the overall mean. Note that for interpretability RSMR is sometimes scaled by the average sample mortality rate. However, this linear transformation does not affect the hospital ranking.

#### b2) ER based on random effects logistic regression

Analogous to the logistic-regression-based excess risk, the excess risk based on the random effects logistic regression model was derived as ${\hat{\text{ER}}}_{h}^{\text{RE}\;\text{Logit}}=O_{h}-{\hat{\bar{E}}}_{h}^{\text{RE}\;\text{Logit}}$.

#### c1) SMR based on fixed effects logistic regression with Firth correction

Both the hierarchical random effects and the non-hierarchical logistic regression approach implicitly rely on the assumption that risk factor and care quality are uncorrelated, i.e. *ρ* = 0. Fixed effects models relax this assumption. In panel econometrics, fixed effects models are routinely applied when there is reason to suspect that observed influence factors are correlated with unobserved, time constant variables [24]. Although the data considered here do not have a time dimension, there is a related structure. While panel data is characterized by multiple time periods per unit [21, 22, 24], our data contains multiple patients nested within one hospitals. To estimate a logistic fixed effects model, we included hospital-specific dummy variables *D*_h*_ = *I*(*h* = h*), h* = 1, …, *H* in the logistic regression model, where *I*(⋅) is the indicator function. The regression equation thus was

$$E\left[Y_{hi}|D_{1},\ldots ,D_{H},X_{hi}\right]=F^{-1}(\sum_{h^{*}=1}^{H}\omega_{h^{*}}\cdot D_{h^{*}}+\alpha_{1}\cdot X_{hi}).$$ (8)

Note that this specification relates to the data generating Eq 1 by *α*_1_ = 1 and *ω*_h*_ = *β*_0_ + *β*_1_*Q*_h*_, h* = 1, …, *H*. The coefficients *ω*_h*_ reflect risk-factor adjusted mortality differences between the hospitals. Since the model treats the hospital-specific dummy variables *D*_h*_ as regressors, Eq 8 is a multiple logistic regression model. Estimation of those models takes correlation between regressors into account [19]. Thus, we expected the assessment of hospital performance based on the fixed effects model to be more robust against correlation between *X*_*hi*_ and *Q*_*h*_.

When estimating the fixed effects model given by Eq 8, we accounted for the small sample bias of the maximum likelihood estimator [25] and potential convergence problems caused by separation [26] by applying Firth correction [17]. Instead of maximizing the ordinary likelihood function *L*(***ω***, *α*_1_) of the logistic regression model, Firth’s logistic regression maximizes a penalized likelihood function *L*(***ω***, *α*_1_) ⋅ |*V*(***ω***, *α*_1_)|^1/2^, where |*V*(⋅)| is the determinant of the Fisher information matrix. Previous studies confirmed that fixed effects logistic regression with Firth correction performed well in related contexts and reported better convergence compared to ordinary logistic regression [11, 27].

Given the parameter estimates obtained from fixed effects logistic regression with Firth correction, the predicted mortality rate of hospital *h* was calculated as ${\hat{E}}_{h}^{\text{FE}\;\text{Logit}}=n_{h}^{-1}\sum_{i=1}^{n_{h}}F^{-1}\left({\hat{\omega }}_{h}+{\hat{\alpha }}_{1}X_{hi}\right)$. The predicted mortality rate of the hospital given the average hospital performance level $\hat{\bar{\omega }}=n_{h}^{-1}\sum_{h=1}^{H}{\hat{\omega }}_{h}$ was derived as ${\hat{\bar{E}}}_{h}^{\text{FE}\;\text{Logit}}=n_{h}^{-1}\sum_{i=1}^{n_{h}}F^{-1}\left(\hat{\bar{\omega }}+{\hat{\alpha }}_{1}X_{hi}\right)$. Analogous to the RSMR based on the random effects model, the fixed effects RSMR then was obtained by

$${\hat{\text{RSMR}}}_{h}^{\text{FE}\;\text{Logit}}=\frac{{\hat{E}}_{h}^{\text{FE}\;\text{Logit}}}{{\hat{\bar{E}}}_{h}^{\text{FE}\;\text{Logit}}}=\frac{\sum_{i=1}^{n_{h}}F^{-1}\left({\hat{\omega }}_{h}+{\hat{\alpha }}_{1}X_{hi}\right)}{\sum_{i=1}^{n_{h}}F^{-1}\left(\hat{\bar{\omega }}+{\hat{\alpha }}_{1}X_{hi}\right)}.$$ (9)

#### c2) ER based on fixed effects logistic regression with Firth correction

Given ${\hat{\bar{E}}}_{h}^{\text{FE}\;\text{Logit}}$, the ER based on fixed effects logistic regression with Firth correction was calculated as ${\hat{\text{ER}}}_{h}^{\text{FE}\;\text{Logit}}=O_{h}-{\hat{\bar{E}}}_{h}^{\text{FE}\;\text{Logit}}$.

### Adequacy of the hospital performance assessment

Following [15], the adequacy of the hospital performance estimations was assessed by the proportion of hospitals correctly classified into quintiles according to their true care qualities *Q*_*h*_.

### Scenarios

The performance of the measures described above was assessed in multiple scenarios, which differed due to variations of specific parameters relative to a baseline scenario (Table 1). The baseline scenario assumed a setting with $n=\sum_{h=1}^{H}n_{h}=10,000$ patients treated in *H* = 200 hospitals. As outlined above, the number of patients per hospital was distributed according to Fig 2. The average mortality rate in the baseline scenario was set to *p* = 20%. Hospital care quality was distributed as $Q_{h}\overset{i.i.d.}{∼}\text{Beta}(6,6)$, i.e. symmetrically with most care quality values concentrated at intermediate levels (see Fig 1). The odds ratio of care quality was set to *OR* = 0.5. For the baseline mortality rate of 20%, this implied a mortality rate difference of 11 percentage points between the highest and the lowest care quality. The hospital-specific and the patient-specific standard deviations of *X*_*hi*_ were set to *η* = 0.2 and *σ* = 0.6, respectively. This resulted in average pseudo-R-squared values of approximately 0.1 when applying logistic regression to the simulated data. Another parameter introduced to the simulations was the minimum number of patients simulated for each hospital $n_{h}^{\text{min}}$. In the baseline scenario, $n_{h}^{\text{min}}=15$, implying that no considered hospital treated less than 15 patients. Variations of this parameter were used to investigate the robustness of the measures with respect to the presence of hospitals with small volumes. Given this set of parameter values, correlations between risk factor and care quality between -0.8 and 0.8 were simulated. Following related studies [11, 15], Monte Carlo estimates for each parameter constellation were based on 1,000 draws.

**Table 1:** Baseline parameter values.

Parameter(s)	Value(s)	Parameter(s)	Value(s)
*ρ*	{−0.8, −0.6, −0.3, 0, 0.3, 0.6, 0.8}	*q*_1_ = *q*_2_	6
*n*	10,000	*η*	0.2
*H*	200	*σ*	0.6
*OR*	0.5	$n_{h}^{\text{min}}$	15

## Simulation results

**Fig 3:**
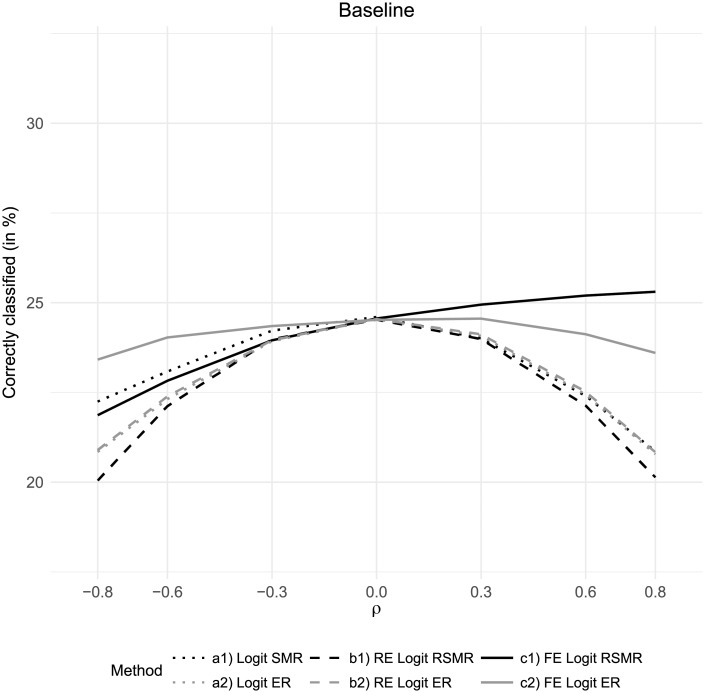
Baseline results: Percent of hospitals correctly classified in to quintiles by correlation between risk factor and care quality.

Without correlation between risk factor and care quality (i.e. *ρ* = 0), all considered methods resulted in similarly high proportions of hospitals correctly classified into quintiles in the baseline scenario (Fig 3). All measures based on logistic regression and random effects logistic regression performed worse when either positive or negative correlation between risk factor and care quality was induced. Particularly the RSMR based on random effects logistic regression was distorted by correlation. With regard to the fixed-effect-based estimations, there were notable differences between the performance of RSMR and ER. While the RSMR based on fixed effects logistic regression outperformed the other approaches in scenarios with high positive correlation between risk factor and care quality, it performed even worse than the SMR based on simple logistic regression in scenarios with negative correlation. In these cases, ER based on fixed effects logistic regression showed the best classification results. The fixed-effects-based ER also outperformed all measures based on logistic regression and random effects logistic regression in case of positive correlation between risk factor and care quality. Thus, the results of the baseline scenario indicated that ER based on fixed effects logistic regression was most robust against correlation between risk factor and care quality.

**Fig 4:**
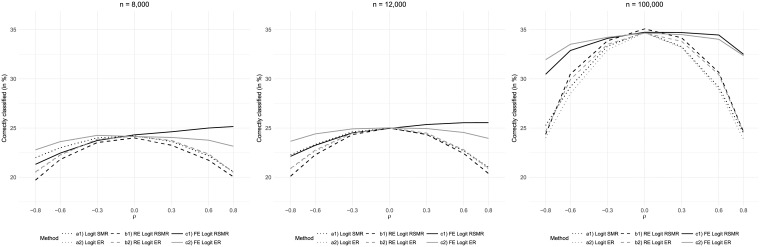
Results for variations of total sample size *n*.

Holding the other parameter values of the baseline scenario constant, we also assessed performances for different sample sizes (Fig 4). All measures showed better classification results when the overall number of patients was increased. However, larger sample sizes did not reduce the distortion of the measures based on logistic regression and random effects logistic regression caused by correlation between risk factor and care quality as both positive and negative correlations resulted in worse classification results. These measures were almost always outperformed by RSMR and ER based on fixed effects logistic regression. The only exception was the slightly better classification result obtained by RSMR based on random effects logistic regression in the scenario characterized by 100,000 patients and the absence of correlation between risk factor and care quality (*ρ* = 0).

**Fig 5:**
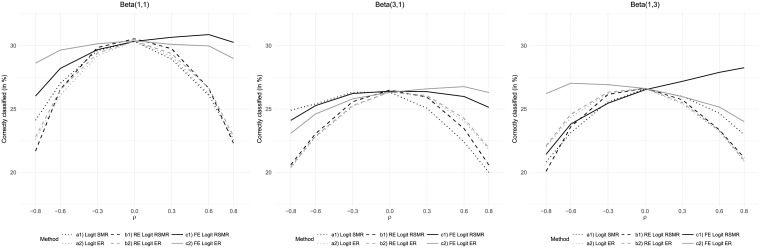
Results for variations of the distribution of care quality *Q*_*h*_.

The results of the hospital performance assessment were found to depend on the distribution of hospital-care quality (Fig 5). A uniformly distributed care quality (Beta(1,1)) generally led to better classification results compared to the baseline scenario (Beta(6,6)). Given a left-skewed distribution (Beta(3,1)), the fixed-effects-based measures performed better than the other measures for positive correlation between risk factor and care quality but slightly worse than the logistic-regression-based SMR for high negative correlation. The assumption of a right-skewed distribution (Beta(1,3)) resulted in a clear dominance of the fixed-effects-regression-based SMR in case of positive correlation and of fixed-effects-regression-based ER in case of negative correlation. The latter also performed better than all measures based on simple logistic regression or random effects regression in case of positive correlation.

**Fig 6:**
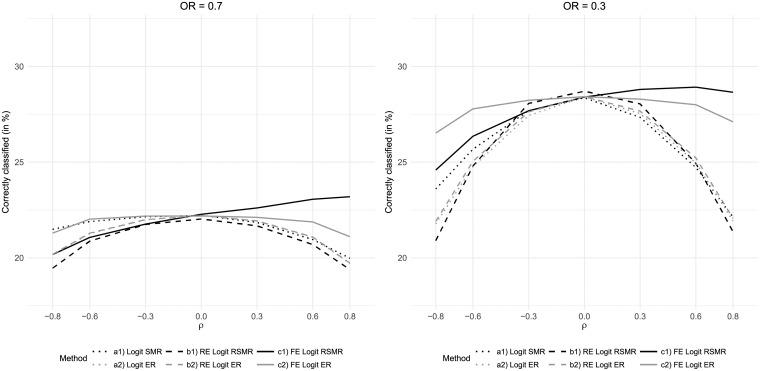
Results for variations of the odds ratio of care quality *OR*.

A reduced influence of care quality on the outcome as induced by an increase in the odds ratio of care quality to 0.7 resulted in worse classification results for all considered measures (Fig 6). The fixed-effects-based measures remained dominant for positive correlations between risk factor and care quality. For negative correlations, the best results were obtained from ER based on fixed effects regression and SMR based on simple logistic regression, which differed only slightly. Inducing greater mortality differences between high-quality and low-quality hospitals by reducing the odds ratio to 0.3 generally led to better classification results. However, there was also an increase in the distortion due to correlation between risk factor and care quality of those measures not based on fixed effects regression. Again, ER based on fixed effects regression was found to be most robust against both positive and negative correlation.

**Fig 7:**
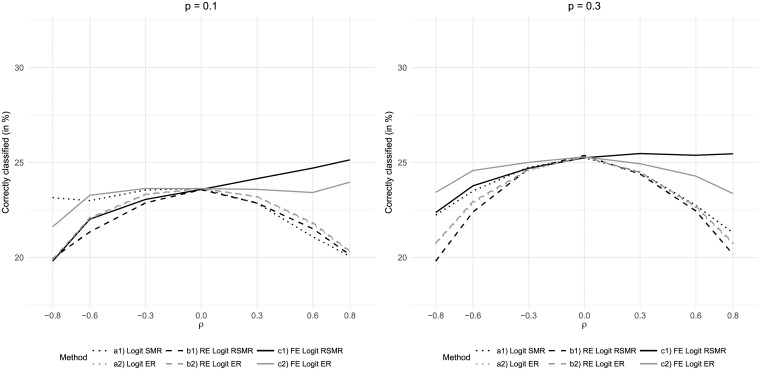
Results for variations of the population average mortality rate *p*.

A reduction of the population average mortality rate from 20% to 10% was associated with a lower proportion of correctly classified hospitals (Fig 7). While the patterns of the classification results qualitatively remained stable, particularly the RSMR-based measures performed worse for strong negative correlations between risk factor and care quality. In most of these scenarios, ER based on fixed effects logistic regression performed best. The fixed-effects-based measures further dominated when positive correlation between risk factor and care quality was induced. Increasing the population average mortality rate to 30% further increased the dominance of the fixed-effects-regression-based measures.

**Fig 8:**
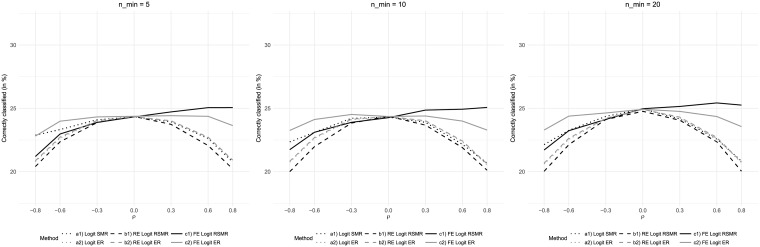
Results for variations of the minimum number of patients per hospital $n_{h}^{\text{min}}$.

Varying the minimum number of patients per hospital did not affect the general patterns observed in previous scenarios (Fig 8). However, particularly the performance of fixed-effects-based measures improved when the number of patients per hospital was increased.

## Discussion

In empirical assessments, hospital performance may be correlated with patient-specific risk factors. Better performing hospitals may treat sicker patients than hospitals with worse performance (or vice versa). Such correlation may also arise when the risk adjustment includes comorbidities that had not been present on admission. These issues are neglected by many common approaches to risk adjustment. Against that background, this study assessed the impact of correlation between hospital performance and risk factors on the adequacy of hospital rankings based on different methods and measures for binary outcomes.

The results of Monte Carlo simulations highlighted that ignoring such correlation may lead to severe bias in the performance assessment. The results for the SMRs/RSMRs and ERs based on logistic regression and random effects logistic regression showed that these approaches generally performed worse when either positive or negative correlation between care quality and risk factor was induced. In contrast, measures based on fixed effects logistic regression with Firth correction were more robust to such correlation. This was particularly true for the logistic-regression-based excess risk, which proved to be most robust against both positive and negative correlation between care quality and risk factor. In scenarios without correlation, all considered methods showed similar performance.

### Strengths and limitations

Based on a simple simulation setup, this study contributes to the sparse literature on fixed effects approaches in the context of hospital performance measurement [11, 27–30] by highlighting the effects of correlation between hospital performance and risk factors on hospital rankings. The comparison of multiple methods and measures is one of the main strengths of the present analysis.

As a main result, measures based on fixed effects logistic regression proved to be relatively robust against correlation between risk factors and care quality. Estimation of fixed effects models is subject to several problems. One problem is the small sample bias of the maximum likelihood estimator of the logistic regression model, which may be substantial in magnitude [25]. Furthermore, the outcome of all or some patients may be perfectly predicted by covariates, particularly by the hospital dummies. This phenomenon is known as separation and may cause severe bias and convergence problems [26]. Separation is particularly likely if the dataset includes hospitals with a small number of patients. However, the results in this paper indicate that these issues can be addressed effectively by applying Firth correction, which is consistent with the findings of [17].

Following the methodology of CMS [10], the estimation of random effects RSMR and ER was based on a model that includes a random intercept at the hospital level. This random intercept accounts for correlation of patient outcomes within a hospital and is crucial for capturing quality differences between hospitals. Future studies may also consider random parameter models to allow for heterogeneous effects of risk factors on patient outcomes [20, 22]. Furthermore, risk adjustment applications may include multiple hospitals over several time periods and be subject to unobserved spatially shared risk factors. While accounting for temporal and spatial correlation is beyond the scope of the present study, using appropriate modeling approaches [31–34] would be a promising route for future research.

Another general limitation is that hospital performance is unobservable in real-world applications. Hence, empirical examination of advantages of fixed effects approaches for specific datasets is not feasible. On average, however, our simulations reveal that particularly fixed-effects-logistic-regression-based ER outperforms approaches based on logistic regression and random effects logistic regression in most scenarios. Although many relevant scenarios have been covered in this simulation study, there may be other interesting scenarios that have not been considered here. As one limitation, this study did not examine the effects of confounding due to omitted relevant risk factors. Furthermore, the generalizability of the results to other outcome types and statistical models is open for exploration. As has been demonstrated in related contexts [23, 31, 32, 34, 35], the use of alternative approaches to logistic regression could also improve statistical modeling of hospital mortality. These topics could be addressed by future research.

### Practical implications

The results of this study indicate that hospital quality indicators based on simple logistic regression and random effects logistic regression have to be interpreted with caution. These approaches may be severely biased when there is correlation between hospital performance and risk factors. Particularly ER based on fixed effects logistic regression with Firth correction was more robust to such correlation. Since we found no relevant differences between methods in the absence of correlation, ER based on fixed effects logistic regression with Firth correction should always be considered when the objective is to rank hospitals according to their performance.

## Supporting information

S1 Appendix Taylor approximation of average mortality rate. (PDF)

S1 Script Script file for simulation. (R)

S2 Script Script for figures. (R)

## References

[1] Schmitt J, Schoffer O, Walther F, Roessler M, Grählert X, Eberlein-Gonska M, et al Effectiveness of the IQM peer review procedure to improve in-patient care—a pragmatic cluster randomized controlled trial (IMPRESS): study design and baseline results. Journal of Public Health. 2019 10.1007/s10389-019-01118-9

[2] Krahwinkel W, Schuler E, Liebetrau M, Meier-Hellmann A, Zacher J, Kuhlen R, et al The effect of peer review on mortality rates. Int J for Qual Health Care. 2016;28(5):594–600. 10.1093/intqhc/mzw072 27424326

[3] Faber M, Bosch M, Wollersheim H, Leatherman S, Grol R. Public reporting in health care: How do consumers use quality-of-care information?: A systematic review. Med Care. 2009;47(1):1–8. 10.1097/MLR.0b013e3181808bb5 19106724

[4] Hafner JM, Williams SC, Koss RG, Tschurtz BA, Schmaltz SP, Loeb JM. The perceived impact of public reporting hospital performance data: interviews with hospital staff. Int J for Qual Health Care. 2011;23(6):697–704. 10.1093/intqhc/mzr056 21840943

[5] Lichtman JH, Leifheit EC, Wang Y, Goldstein LB. Hospital Quality Metrics:“America’s Best Hospitals” and Outcomes After Ischemic Stroke. J Stroke Cerebrovasc Dis. 2019;28(2):430–434. 10.1016/j.jstrokecerebrovasdis.2018.10.022 30415916

[6] Reiter A, Geraedts M, Jäckel W, Fischer B, Veit C, Döbler K. Selection of hospital quality indicators for public disclosure in Germany. Z Evid Fortbild Qual Gesundhwes. 2011;105(1):44–48. 10.1016/j.zefq.2010.12.024 21382604

[7] Aylin P, Bottle A, Jen MH, Middleton S, Intelligence F. HSMR mortality indicators. Imperial College Technical Document. 2010.

[8] Newman SC. Biostatistical Methods in Epidemiology. New York: Johne Wiley & Sons, INC; 2001.

[9] Normand SLT, Wolf RE, Ayanian JZ, McNeil BJ. Assessing the accuracy of hospital clinical performance measures. Med Decis Making. 2007;27(1):9–20. 10.1177/0272989X06298028 17237448

[10] Centers for Medicare & Medicaid Services (CMS). Measure Methodology; 2019. Available from: https://www.cms.gov/Medicare/Quality-Initiatives-Patient-assessment-Instruments/HospitalQualityInits/Measure-Methodology.html.

[11] Varewyck M, Goetghebeur E, Eriksson M, Vansteelandt S. On shrinkage and model extrapolation in the evaluation of clinical center performance. Biostatistics. 2014;15(4):651–664. 10.1093/biostatistics/kxu019 24812420 PMC4173104

[12] Glance LG, Osler T, Shinozaki T. Effect of varying the case mix on the standardized mortality ratio and W statistic: a simulation study. Chest. 2000;117(4):1112–1117. 10.1378/chest.117.4.1112 10767249

[13] Kahn JM, Kramer AA, Rubenfeld GD. Transferring critically ill patients out of hospital improves the standardized mortality ratio: a simulation study. Chest. 2007;131(1):68–75. 10.1378/chest.06-0741 17218558

[14] Rosenthal GE, Shah A, Way LE, Harper DL. Variations in standardized hospital mortality rates for six common medical diagnoses: implications for profiling hospital quality. Med Care. 1998;36(7):955–964. 10.1097/00005650-199807000-00003 9674614

[15] Ryan A, Burgess J, Strawderman R, Dimick J. What is the best way to estimate hospital quality outcomes? A simulation approach. Health Serv Res. 2012;47(4):1699–1718. 10.1111/j.1475-6773.2012.01382.x 22352894 PMC3401406

[16] Statistische Ämter des Bundes und der Länder; 2019. Available from: https://www.forschungsdatenzentrum.de/en/health/drg.

[17] Firth D. Bias reduction of maximum likelihood estimates. Biometrika. 1993;80(1):27–38. 10.1093/biomet/80.1.27

[18] Statistische Ämter des Bundes und der Länder. Verzeichnis der Krankenhäuser und Vorsorge- und Rehabilitatsionseinrichtungen in Deutschland; 2019. Available from: https://www.destatis.de/DE/Themen/Gesellschaft-Umwelt/Gesundheit/Krankenhaeuser/_inhalt.html#sprg234206.

[19] Hosmer DW Jr, Lemeshow S, Sturdivant RX. Applied logistic regression. vol. 398 John Wiley & Sons; 2013.

[20] Chen F, Chen S. Injury severities of truck drivers in single-and multi-vehicle accidents on rural highways. Accid Anal Prev. 2011;43(5):1677–1688. 10.1016/j.aap.2011.03.026 21658494

[21] Chen F, Chen S, Ma X. Crash Frequency Modeling Using Real-Time Environmental and Traffic Data and Unbalanced Panel Data Models. Int J Environ Res Public Health. 2016;13(6):609 10.3390/ijerph13060609 PMC4924066 27322306

[22] Chen F, Chen S, Ma X. Analysis of hourly crash likelihood using unbalanced panel data mixed logit model and real-time driving environmental big data. J Safety Res. 2018;65:153–159. 10.1016/j.jsr.2018.02.010 29776524

[23] Chen F, Song M, Ma X. Investigation on the Injury Severity of Drivers in Rear-End Collisions Between Cars Using a Random Parameters Bivariate Ordered Probit Model. Int J Environ Res Public Health. 2019;16(14):2632 10.3390/ijerph16142632 PMC6678079 31340600

[24] Wooldridge JM. Econometric analysis of cross section and panel data. Cambridge: MIT Press; 2010.

[25] Peduzzi P, Concato J, Kemper E, Holford TR, Feinstein AR. A simulation study of the number of events per variable in logistic regression analysis. J Clin Epidemiol. 1996;49(12):1373–1379. 10.1016/s0895-4356(96)00236-3 8970487

[26] Mansournia MA, Geroldinger A, Greenland S, Heinze G. Separation in logistic regression: causes, consequences, and control. Am J Epidemiol. 2017;187(4):864–870. 10.1093/aje/kwx299 29020135

[27] Varewyck M, Vansteelandt S, Eriksson M, Goetghebeur E. On the practice of ignoring center-patient interactions in evaluating hospital performance. Stat Med. 2016;35(2):227–238. 10.1002/sim.6634 26303843 PMC5049670

[28] Glance LG, Dick A, Osler TM, Li Y, Mukamel DB. Impact of changing the statistical methodology on hospital and surgeon ranking: the case of the New York State cardiac surgery report card. Med Care. 2006;44(4):311–319. 10.1097/01.mlr.0000204106.64619.2a 16565631

[29] Kalbfleisch JD, Wolfe RA. On monitoring outcomes of medical providers. Stat Biosci. 2013;5(2):286–302. 10.1007/s12561-013-9093-x

[30] Moran JL, Solomon PJ, ANZICS Centre for Outcome and Resource Evaluation (CORE) of the Australian and New Zealand Intensive Care Society (ANZICS). Fixed Effects Modelling for Provider Mortality Outcomes: Analysis of the Australia and New Zealand Intensive Care Society (ANZICS) Adult Patient Data-Base. PLoS ONE. 2014;9(7):e102297 10.1371/journal.pone.0102297 25029164 PMC4100889

[31] Zeng Q, Wen H, Huang H, Abdel-Aty M. A Bayesian spatial random parameters Tobit model for analyzing crash rates on roadway segments. Accid Anal Prev. 2017;100:37–43. 10.1016/j.aap.2016.12.023 28088033

[32] Zeng Q, Wen H, Huang H, Pei X, Wong S. Incorporating temporal correlation into a multivariate random parameters Tobit model for modeling crash rate by injury severity. Transportmetrica A: Transport Science. 2018;14(3):177–191. 10.1080/23249935.2017.1353556

[33] Zeng Q, Gu W, Zhang X, Wen H, Lee J, Hao W. Analyzing freeway crash severity using a Bayesian spatial generalized ordered logit model with conditional autoregressive priors. Accid Anal Prev. 2019;127:87–95. 10.1016/j.aap.2019.02.029 30844540

[34] Zeng Q, Guo Q, Wong S, Wen H, Huang H, Pei X. Jointly modeling area-level crash rates by severity: a Bayesian multivariate random-parameters spatio-temporal Tobit regression. Transportmetrica A: Transport Science. 2019;15(2):1867–1884. 10.1080/23249935.2019.1652867

[35] Zou G. A Modified Poisson Regression Approach to Prospective Studies with Binary Data. Am J Epidemiol. 2004;159(7):702–706. 10.1093/aje/kwh090 15033648

