# Supplementary material for: Ranking hospitals when performance and risk factors are correlated: A simulation-based comparison of risk adjustment approaches for binary outcomes
Source: PLoS One. 2019 Dec 4;14(12):e0225844. doi: 10.1371/journal.pone.0225844 (PMC6892499; doi:10.1371/journal.pone.0225844)
Supplement: S1 Appendix — (PDF) [file pone.0225844.s001.pdf]

Supporting information:
Ranking hospitals when performance and risk factors are correlated: a simulation-based comparison of risk adjustment approaches for binary outcomes

Martin Roessler[1*], Jochen Schmitt[1], Olaf Schoffer[1]

1 Zentrum für Evidenzbasierte Gesundheitsversorgung, Universitätsklinikum und Medizinische Fakultät Carl Gustav Carus an der Technischen Universität Dresden, Dresden, Germany

* martin.roessler@uniklinikum-dresden.de

Taylor approximation of average mortality rate

The data generation process specified the probability of observing the outcome $Y_{hi} = 1$ as

$$E[Y_{hi}|Q_h, X_{hi}] = P(Y_{hi} = 1|Q_h, X_{hi}) = F^{-1}(\beta_0 + \beta_1 Q_h + X_{hi}), \tag{1}$$

where X_{hi} was generated using

$$X_{hi} = \gamma \cdot Q_h + a_h + \varepsilon_{hi}, \tag{2}$$

$Q_h \overset{i.i.d.}{\sim} \text{Beta}(q_1, q_2)$, $a_h \overset{i.i.d.}{\sim} N(0, \eta^2)$, $\varepsilon_{hi} \overset{i.i.d.}{\sim} N(0, \sigma^2)$.

As noted in the paper, correlation between care quality Q_h and risk factor X_{hi} changes the population average mortality rate, which may, by the law of iterated expectations, be derived as

$$E[Y_{hi}] = E[E[Y_{hi}|Q_h, X_{hi}]]. \tag{3}$$

Since this parameter is crucial for the adequacy of the performance estimation, we used Taylor approximation of Eq 3 to keep $E[Y_{hi}]$ approximately constant. Applying fist-order Taylor expansion of $E[Y_{hi}|Q_h, X_{hi}]$ around the point y and taking expectations on both sides of the equation yields

$$E[Y_{hi}] \approx E[F^{-1}(y) + F^{-1'}(y) \cdot (\beta_0 + \beta_1 Q_h + X_{hi} - y)] \tag{4}$$

Choosing $y = F(E[Y_{hi}])$ and solving for β_0 yields

$$\beta_0 \approx F(E[Y_{hi}]) - \beta_1 E[Q_h] - E[X_{hi}] = F(E[Y_{hi}]) - \frac{q_1}{q_1 + q_2}(\beta_1 + \gamma). \tag{5}$$

Hence, we used Eq 5 to determine the value β_0 in order to approximate a specific population average mortality rate $E[Y_{hi}]$ in each simulated scenario.
